# Downregulated ARID1A by miR-185 Is Associated With Poor Prognosis and Adverse Outcomes in Colon Adenocarcinoma

**DOI:** 10.3389/fonc.2021.679334

**Published:** 2021-08-02

**Authors:** Salem Baldi, Hassan Khamgan, Yuanyuan Qian, Han Wu, Zhenyu Zhang, Mengyan Zhang, Yina Gao, Mohammed Safi, Mohammed Al-Radhi, Yun-Fei Zuo

**Affiliations:** ^1^Department of Clinical Biochemistry, College of Laboratory Diagnostic Medicine, Dalian Medical University, Dalian, China; ^2^Department of Molecular Diagnostics and Therapeutics, University of Sadat City, Sadat, Egypt; ^3^Department of Oncology, First Affiliated Hospital of Dalian Medical University, Dalian, China; ^4^Department of Urology, Second Affiliated Hospital of Dalian Medical University, Dalian, China

**Keywords:** AT-rich interaction domain 1A (ARID1A), miR-185-5P, gene expression, posttranscriptional regulation, The Cancer Genome Atlas (TCGA), immune cell infiltration

## Abstract

AT-rich interaction domain 1A (ARID1A) is a tumor suppressor gene that mutates in several cancer types, including breast cancer, ovarian cancer, and colorectal cancer (CRC). In colon adenocarcinoma (COAD), the low expression of ARID1A was reported but the molecular reason is unclear. We noticed that ARID1A low expression was associated with increased levels of miR-185 in the COAD. Therefore, this study aims to explore ncRNA-dependent mechanism that regulates ARID1A expression in COAD regarding miR-185. The expression of ARID1A was tested in COAD cell line under the effect of miR-185 mimics compared with inhibitor. The molecular features associated with loss of ARID1A and its association with tumor prognosis were analyzed using multi-platform data from The Cancer Genome Atlas (TCGA), and gene set enrichment analysis (GSEA) to identify potential signaling pathways associated with ARID1A alterations in colon cancer. Kaplan-Meier survival curve showed that a low level of ARID1A was closely related to low survival rate in patients with COAD. Results showed that inhibiting miR-185 expression in the COAD cell line significantly restored the expression of ARID1A. Further, the increased expression of ARID1A significantly improved the prolonged overall survival of COAD. We noticed that there is a possible relationship between ARID1A high expression and tumor microenvironment infiltrating immune cells. Furthermore, the increase of ARID1A in tumor cells enhanced the response of inflammatory chemokines. In conclusion, this study demonstrates that ARID1A is a direct target of miR-185 in COAD that regulates the immune modulations in the microenvironment of COAD.

**Graphical Abstract:**
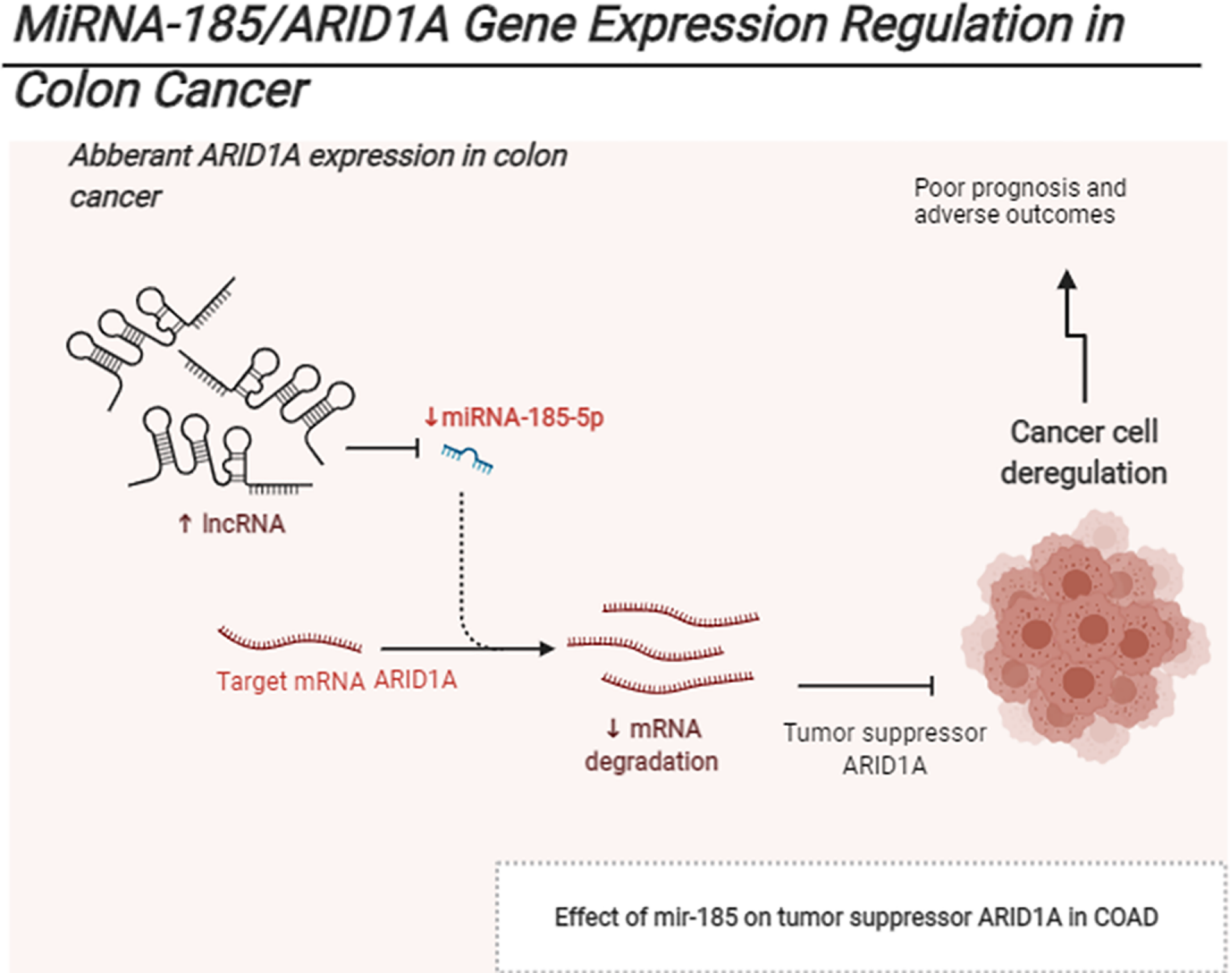
The schematic represents Mir185 controls ARID1A expression in COAD.

## Introduction

Colorectal cancer (CRC) is one of the most prevalent forms of cancer in the world, which is responsible for the most cancer deaths (1). Several genes, including ARID1A, are involved in the pathogenesis of CRC (2). Colon cancer is also one of the most affected cancers in terms of intertumoral heterogeneity caused by immunosuppressive and dysfunctional immune cell subsets. These include M2 macrophage polarization, CD8+ T cells, B cells, and natural killer cells (3). Shen et al. discovered that ARID1A deficiency was associated with decreased mismatch repair protein capacity, increased cancer mutability, and proposed that ARID1A could increase immune checkpoint blockade therapy efficacy (4). Many researchers have demonstrated that tumors can inhibit the function and infiltration of cytotoxic T lymphocytes (CTLs) (5, 6). In this regard, some genes may interact with CTLs and either prevent CTLs infiltration or inhibit the activity of CTLs, thus promoting immune escape and immunotherapy tolerance. Recent studies have reported that ARID1A elevates the expression of programmed cell death ligand 1 (PD-L1) and modulates the immune microenvironment (7). Further, ARID1A deficiency was reported to activate phosphatidylinositol 3-kinase (PI3K)/protein kinase B (AKT) signaling and increased PD-L1 expression in gastric cancer (7). ARID1A is the core subunit of SWItch/Sucrose Non-Fermenting (SWI/SNF), which plays an essential role in DNA transcription, replication, and damage repair. In various types of cancer, chromatin remodeling factor, ARID1A, is frequently mutated and acts as a tumor suppressor (8, 9). Recently, decreased mRNA ARID1A and protein expression have been associated with poor prognosis in gastric cancer (9). In colon cancer, ARID1A protein loss was associated with clinicopathologic features, distant metastases, and poor overall survival (10). However, the mechanism by which ARID1A expression was decreased, other than loss of function mutation, remains unknown.

The post-transcriptional alteration of mRNAs is one of the most well-known mechanisms by which miRNAs regulate their target genes. Although the ARID1A deficiency is widely documented, there has been no research on ARID1A-dependent mRNA expression and post-transcriptional repression by non-coding miRNAs in COAD. To address this gap, we searched for miRNAs-targeted ARID1A in TCGA-based data tools and found a subset of miRNAs that may target ARID1A in colon cancer. We also uncovered the prognosis significance of low ARID1A levels in colon cancer, using RNA sequencing data from the TCGA database. We discovered that ARID1A predicted poor prognosis in colorectal cancer patients.

## Materials and Methods

### Aberration Expression of ARID1A and Its Clinical Significance in COAD

TNMplot provides a comprehensive analysis of RNA sequence expression and Gene Chip data of tumor pan-cancer. We used the pan-cancer analysis page to evaluate the expression level of the ARID1A gene across all tissues in all available normal and tumor RNA Sew data (https://www.tnmplot.com/). We used RNA sequencing and Gene Chip data to determine the differential expression of ARID1A in normal and tumor COAD tissues. The ARID1A expression profile was compared using Gene Chip data from tumor, normal, and metastasis tissues.

### Prognostic Analysis of ARID1A

GEPIA 2 was used to study the prognosis of ARID1A in TCGA COAD (http://gepia2.cancer-pku.cn/#index). A statistical test from the TCGA database was used to analyze the correlation between ARID1A expression and clinical features in COAD patients, including age, gender, tumor stage, pathological stages, and tumor purity. Utilizing USCS Xian TCGA database http://xena.ucsc.edu/., we investigated the association between ARID1A and the standard clinic pathological variables in COAD. We further evaluated ARID1A’s prognostic importance using the previously published GEO dataset and discovered that a lower ARID1A level was correlated with a poor prognosis at a 5% statistical significance level.

### Cell Culture and q PCR

Human colon cancer cell lines HCT116 and LoVo were cultured in Dulbecco’s Modified Eagle medium (DMEM) or RPM1640 with medium 10% fetal bovine serum (FBS) at 37°C with 5% CO_2_. For transfection, HCT 116 and LoVo cells were seeded into 12-well plates and transfected according to the manufacturer’s instructions using Lipofectamine 2000 (GenePharma, China). Cells were isolated after being transfected for 24 to 36 h, and after that, they were processed further. Total RNA was extracted from the cells using TRIzol Reagent [Code AG21102, Accurate Biotechnology (Hunan) Co., Ltd].

cDNA was obtained using kit [Code AG11605, Accurate Biotecnology (Hunan) Co., Ltd, China], which served as a qPCR template, and the qPCR reaction mix was prepared in triplicate. Primers ARID1A Forward” TTATGACAGAGTGAGGACGGAG, and ARID1A Reverse” TGCCTTGGGTGGAGAACTGAT mixed with SYBR green analysis reagent as recommended by the manufacturer (company). GAPDH, a housekeeping gene, was used to normalize gene expression in the same sample. Normalized Ct values were determined using the ΔΔCt method.

### Western Blotting

The total protein derived from cells was extracted using RIPA lysis buffer with PMSF and cocktail protease inhibitors, quantified using the BCA method in lysis buffer (Beijing Solaribo Science & Technology). A total protein was separated using 8% SDS-PAGE and then transferred to a Millipore PVDF membrane. After blocking with 5% non-fat milk in Tris-buffered saline (1× TBS) containing 0.05% Tween-20, the membrane was incubated overnight at 4°C with rabbit antibodies against ARID1A (1:300, CUSABIO TECHNOLOGY), βactin (1:1000, Beijing Zhongshan Biotechnology Co., Ltd). After washing, the membrane was incubated at 37°C for 2 h with a secondary antibody conjugated to horseradish peroxidase (Beijing Solaribo Science & Technology).

### Transcription Regulation of ARID1A

We predicted ARID1A-targeted miRNAs using several Web-based TCGA databases, including Gene Set Cancer Analysis (GSCALite), CanEvolve, and linkedomics. Following that, we used the UALCAN database to detect ARID1A-targeted mir185 expression in COAD. Notably, mir185 was shown to target ARID1A using miRNA mimics and inhibitors. GraphPad software was used to analyze the ARID1A mRNA expression data and to visualize the results. The protein expression was evaluated by SDS-PAGE and Western blotting using the ARID1A antibody (USABIO TECHNOLOGY). The primary antibodies were detected with HRP-conjugated secondary antibodies, and target protein bands were visualized using LAS-500 imaging system. The study was replicated three times to ensure the reliability of the findings. Additionally, Cistrome database-based chip-seq data were used to predict candidate transcription factors that are most likely to bind and regulate ARID1A expression in COAD (http://cistrome.org/db/#/).

### Gene Set Enrichment Analysis of ARID1A Correlated Genes

To further elucidate ARID1A’s tumor-promoting mechanism in COAD, we sequenced 379 colon cancer tissues in linkedOmics database. LinkedOmics is a freely accessible portal that contains multi-omics data from all 32 TCGA cancer types, which includes genetic information such as DNA mutations, gene expression, and chromosome copy number variation (http://www.linkedomics.org/login.php). Cancer Gene and Explorer Pathways were used in conjunction with LinkedOmics to investigate ARID1A-related function using TCGA and GEO gene expression data (https://cgpe.soic.iupui.edu/gsea_result/).

### Correlation Between ARID1A and Immune Cell Infiltration

We studied immune cell infiltration using the TIMER2 tool, which can directly analyze TCGA data and quantify the ratio of immune cells in the tumor and normal samples based on gene expression profiles, copy number, and mutation status. We established a link between the infiltration of sex immune cell types and ARID1A mRNA expression, mutation, and copy number alterations (http://timer.cistrome.org/).

### Association Between CTLs and Clinicopathological Variables

The association between infiltration of tumor immune subsets and clinical outcome was tested using the multivariable Cox proportional hazard model. The analysis was adjusted for standard clinicopathological characteristics, including age, stages, and tumor purity variables, whereas ARID1A expression was used as a confounding factor in the study.

## Results

### Differential Expression of ARID1A

**Figure 1:**
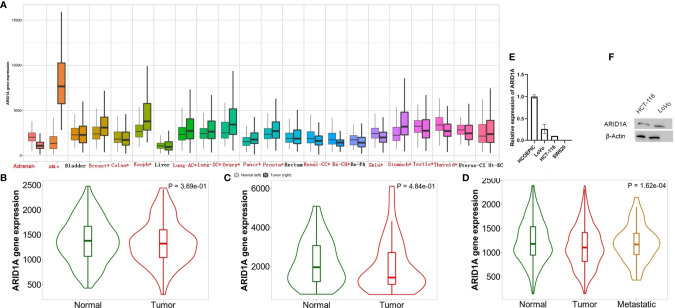
Differential expression of ARID1A. **(A)** Differential ARID1A expression analysis across several cancer types (significant differences by the Mann-Whitney U test are marked with red*). Left, normal; right, tumor); **(B)** ARID1A expression include paired tumor and adjacent normal tissues from Gene chip data; **(C)** ARID1A expression include paired tumor and adjacent normal tissues from RNA-Seq data; **(D)** Significant different expression of ARID1A through metastases, normal, and tumors; **(E–F)** Experimental verification of ARID1A mRNA and protein levels in colon cancer cell line using qPCR and western blot, respectively.

In recent years, researchers have developed online approaches, including GEPIA2, TNMplot, and Linkedomics, to analyze and visualized TCGA data. ARID1A level changes across several TCGA tissues, including COAD, were first evaluated using the TNMplot platform (**Figure 1A**). Analyzing the RNA-seq and Gene chip data provided in the TNMplot database revealed the significant downregulation of ARID1A in primary COAD tissues (**Figures 1B, C** **)**. Further, we analyzed gene expression of ARID1A in COAD metastases and found that ARID1A had a lower expression level in metastases compared to normal (**Figure 1D**). To verify the RNA sequencing results, we detected ARID1A expression in cell lines and tissues of COAD using quantitative RT-PCR and Western blot. Consistently, ARID1A rnRNA and protein levels were significantly downregulated in COAD cell lines, including HCT116 and LoVo cell lines, than HCOEPIC normal cell line (**Figures 1E, F**).

### The Prognostic Implication of ARID1A in COAD

**Figure 2:**
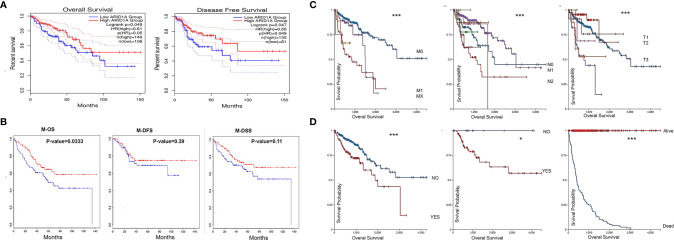
COAD patients with low ARID1A mRNA levels had a poorer prognosis and predictive value in the pathological stages of COAD. **(A)** Prognostic value of ARID1A in primary colon adenocarcinoma using Kaplan-Meier plotter from GEPIA2, OS, Overall survival, DFS, Disease free survival; **(B)** Prognostic value of ARID1A in metastases using Kaplan-Meier plotter from CanEvolve: M-OS, Metastases overall survival, M-DSS, disease-specific survival, and M-DFS, Metastases disease-free survival; **(C)** Pathologic M, pathologic N, and pathologic T, respectively; **(D)** Lymphatic invasion, microsatellite instability, and Vital status, respectively. *p < 0.05 and ***p < 0.001.

The predictive value of ARID1A in COAD was assessed using GEPIA2. Based on mRNA expression data provided in GEPIA2, we found that COAD patients with low ARID1A levels had short overall survival and disease-free survival (**Figure 2A**). To confirm this, we analyzed previously published GSE17536 and found that ARID1A’s low level was also significantly associated with worse OS in COAD metastases. The low level of ARID1A was associated with poor disease-specific survival and disease-free survival, although the p-value was not significant (**Figure 2B**).

### ARID1A Expression in Colon Cancer Tissue Samples and Its Relationship With the Pathological Stages

We further investigated the clinicopathological role of ARID1A expression in colon cancer. ARID1A correlates with some clinicopathological parameters, including age, gender, tumor stage, pathological stages, and tumor purity. UCSC Xina http://xena.ucsc.edu/ was launched to analyze the correlation between ARID1A level and selected pathological features of TCGA COAD patients. Low ARID1A was reduced in the advanced TNM stage (**Figure 2C**) and associated with worse OS in patients with lymphatic invasion, no microsatellite instability, and death status, respectively (**Figure 2D**). As shown in **Figure 2**, the results indicate that the ARID1A gene expression profile predicts recurrence and death in colon cancer patients.

### ARID1A Regulation Network Analysis

**Figure 3:**
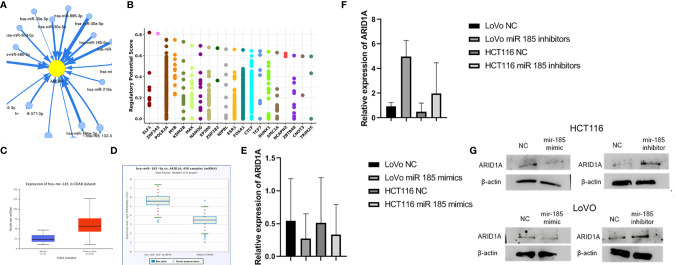
Mir-185 downregulates ARID1A in colon cancer. **(A)** ARID1A can be controlled by mir-185-5p; **(B)** Transcription factors that regulate ARID1A expression from Cistrome (Chip-Seq data); **(C)** Mir-185 is overexpressed in COAD; **(D)** ARID1A is negatively correlated with mir-185-5p in COAD; **(E, F)** Mir185 mimics and inhibitors results detected by qPCR; **(G)** Mir185 mimics and inhibitors results detected by western blot.

Mir-185 has been implicated in regulating tumor-associated genes in various cancer types, but its function in ARID1A suppression is unknown. We first used GSCALite tool to identify the ARID1A miRNAs network in COAD. The data represented in **Figure 3A** show that mir185-5p targets ARID1A reported by the GSCALite tool. We also used ChIP-seq data and identified transcription factors that might posttranscriptionally regulate ARID1A expression in COAD (**Figure 3B**).

Expression analysis of mir-185 based on the TCGA database revealed mir185 was upregulated in COAD tissue. As **Figure 3C** shows, there is a significant difference (p = 0.00026293) between the two groups. Starbase v.3 provides miRNA-associated genes correlation using this function; therefore, a negative correlation between mRNA ARID1A level and mir-185 in COAD was detected in **Figure 3D**.

HCT 116 and LoVo cells were transfected with miR-185 and checked for the impact on ARID1A levels. We found that miR-185-5p mimics significantly reduced the ARID1A level compared with NC, and the reduction was repealed by miR-185-5p inhibitors as evidenced by qRT-PCR (**Figures 3E, F**). Additionally, we performed a Western blot analysis to examine the ARID1A protein level. The results showed that miR-185 mimics and inhibitors significantly decreased and increased ARID1A protein levels, respectively (**Figure 3G**). The most striking result to emerge from the data in **Figure 3** is that ARID1A is a direct target of mir185-5p and is an oncogenic involved in COAD tumor progression, which further confirmed RNA-seq results.

### Determining the Function of ARID1A in Colon Cancer

**Figure 4:**
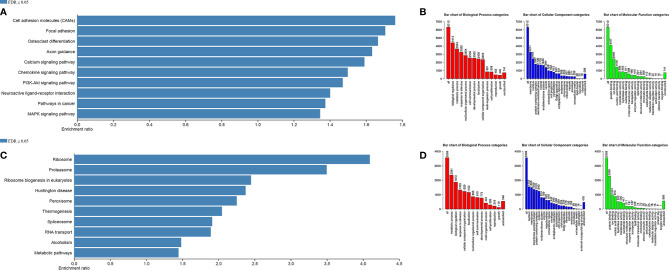
Gene Set Enrichment Analysis. **(A, B)** KEGG pathways and Gene Ontology of significant positively correlated genes, respectively; **(C, D)** KEGG pathways and Gene Ontology of significant negatively correlated genes, respectively.

To understand the function of ARID1A in COAD, we utilized available TCGA sequencing data using LinkedOimcs online tool. The association results and top 50 positively and negatively correlated genes are shown in **Supplementary Figure 1**. The KEGG and Gene Ontology analyses were performed to identify biological and phenotypic pathways associated with the ARID1A gene. Interestingly, GSEA analysis of positively associated genes was enriched in multiple cancer-related pathways, including (1) cell adhesion molecules, (2) chemokine signaling pathway, (3) PI3K-Akt signaling pathways, (4) MAPK signaling pathway, (5) pathways in cancer (**Figures 4A, B**). However, the gene ontology annotation and KEGG signaling pathways of negatively correlated genes were more relevant with metabolic pathways, RNA transport, ribosome, spliceosome, and Huntington disease. (**Figures 4C, D**).

### Correlation Between ARID1A and Infiltration of Immune Cells

**Figure 5:**
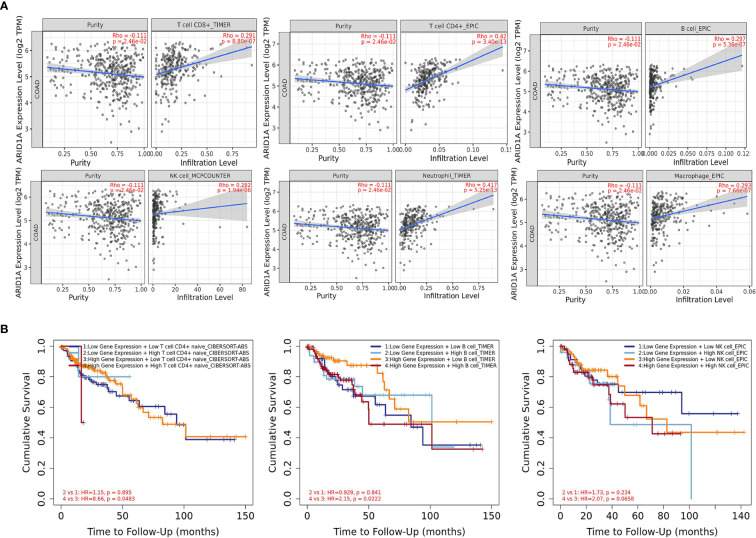
The correlation between ARID1A and immune cell infiltration. **(A)** The significant positive correlation between ARID1A and subtypes of immune cells (TIMER); **(B)** Kaplan-Meier curves for the immune infiltrates and ARID1A Expression. The correlation between ARID1A expression and the abundance of CD4+ T cells, B cells, and natural killer cells, respectively (TIMER).

It was interesting to note the abovementioned findings; hence, we intended to establish whether ARID1A modulates the tumor microenvironment. Therefore, the second set of analyses examined the impact of ARID1A on the immune infiltration level in the COAD microenvironment. We observed statistically significantly higher immune cell counts in tumors with ARID1A gene expression across various cancers. In particular, there was a strong positive correlation of CD8+, CD4+ T cells, B cells, NK cells, as well as macrophages and neutrophils with ARID1A expression level in COAD. However, all associations were adjusted for tumor purity and remained statistically significant (**Figure 5A**).

During the tumor progression and the development of cancer cells, immune cells interact by multiple genes and pathways. Hence, we gain insight into predicting the influence of infiltrated CTLs on the clinical prognosis of patients and explore whether the interaction between the expression level of the ARID1A gene and the level of CTLs impacts the patient’s survival. The CTLs cells were separated into four groups according to ARID1A expression and immune cell infiltrations while using the median as the cutoff in the analysis. Furthermore, hazard ratio and p-value for Cox regression indicated that CTLs infiltration was significantly associated with increased risk of COAD patients. Cox regression analysis showed the impact of ARID1A on patients’ outcomes (**Figure 5B**).

ARID1A shows a positive correlation with a wide range of chemokines and immune cell biomarkers, chemokines, receptors, and inhibitors in colon cancer (**Supplementary Table 1**). These results indicate that ARID1A may affect the immune response of colon cancer. We found no statistically significant tumor-infiltrating lymphocytes (TIL) counts in tumors harboring CNV or somatic oncogenic ARID1A mutations than tumors that were not amplified or mutated in the ARID1A gene.

## Discussion

Researchers use the TCGA database to analyze mRNA expression, methylation, mutation, and copy number alteration (CNAs) (11–15). Several studies reported ARID1A loss expression in CRC, but the mechanisms that lead to ARID1A silencing are still unknown. ARID1A mutations are the main focus of the existing studies. We found that ARID1A is significantly downregulated in COAD tumor malignant than adjacent normal tissues. These results reflect those of Erfani et al. (16), who also found that ARID1A showed no or low expression in CRC (16). Compared with primary colorectal tumors and normal intestinal mucosa, ARID1A exhibits higher expression in metastatic lesions, but its low expression was associated with worse survival in metastases. When primary tumors are metastatic, novel transcripts can be generated at different levels, or novel mRNAs are synthesized at differing amounts in metastases (17). The ARID1A loss expression was detected in colon cancer samples and was not associated with overall, disease-specific, or recurrence-free survival (18). However, the current report found a significant correlation between loss of ARID1A expression and primary colon and metastases cancer stages, suggesting that loss of ARID1A expression may drive tumor formation, invasion, and tumor differentiation (19). Furthermore, ARID1A loss was related to clinicopathological parameters, including clinical and pathological stages as well as survival status. This result consistent with the previously published studies (10, 18). This complete concordance of the lower mRNA and protein loss of ARID1A in COAD strengthen the evidence that ARID1A is a major pathological factor in COAD pathogenesis, significantly associated with adverse outcome. Thus, ARID1A may serve as a prognostic predictor in COAD.

In the following two respects, the mechanism of downregulation of ARID1A in CRC was mainly explained. Firstly, we discovered that miR-185 binds to the ARID1A 3’ UTR, validating databases findings that miR-185 targets and regulates ARID1A expression. Additionally, miR-185 expression was inversely associated with ARID1A expression in CRC tissues. Similarly, gastric cancer samples exhibit absents or weak ARID1A protein expression even though there were no detectable ARID1A mutations (20). TFs could either promote or inhibit gene expression post-transcriptionally. Therefore, we secondly identified a cluster of TFs that can interact and regulate endogenous ARID1A expression in COAD. Further studies need to be conducted on the current topic.

Several recent studies suggested that tumor microenvironmental cells play an essential role in the occurrence and progression of various cancers, including colon cancer (21). There was a significant positive correlation between ARID1A expression and infiltration of macrophages, neutrophils, NK, B cells, CD8+, and CD4+ immune cells. Nevertheless, ARID1A CNAs were not associated with immune cell infiltrations in our TCGA cohort (22–24).

The degree to which a gene impacts CTLs is reflected in the prediction of CTLs on patient clinical prognosis. Cox regression analysis showed that low CTL levels in patients with high ARID1A expression levels indicate a better prognosis. Whereas in patients with low ARID1A expression levels, high CTL levels indicate a worse prognosis. These findings are supported by another research that shows that increasing levels of ARID1A associated with a decrease in T-cell levels promotes cancer cell survival (25). We used GSEA to uncover the underlying regulatory processes leading to CTLs differences between the two groups. ARID1A have been found to utilize chemokine signaling pathway to regulate CTLs in COAD. This analysis provides preliminary data on the role of ARID1A in CTL infiltration. To learn more about the effects of ARID1A on immunological infiltration, we evaluated previously described biomarkers of major immune cell types (15, 26) and their association with ARID1A levels (**Table 1**). Reportedly, the immunoinhibitory molecules attach to the PD-1 receptor on the surface of CD8+ T cells to stop their function. Hence these molecules view immunotherapy resistance as the primary cause (15). While ARID1A deficiency enhanced PDL-1 expression (24), the findings of this study generally validated prior research in this field and proved that ARID1A deletion may influence immunity-related pathways. The altered tumor microenvironment immune cell populations in ARID1A-induced T-lymphocyte dysfunction have not been tested in the current report. However, further studies need to be elucidated.

**Table 1:** Correlation analysis between ARID1A and gene biomarkers of immune cells in COAD (TIMER).

Immune cell types	Biomarkers	Correlation	P value
**T cell (general)**	CD3E	0.245573	5.45E-07
	CD2	0.179266	0.000283
**CD8+ T cell**	CD8A	0.176948	0.00034
**B cell**	CD79A	0.147069	0.002974
**Monocyte**	CD86	0.200761	4.62E-05
	CD115(CSF1R)	0.328745	1.10E-11
**M1 macrophage**	COX2(PTGS2)	0.173564	0.000443
**M2 macrophage**	CD163	0.272391	1.05E-10
	VSIG4	0.117725	0.017642
	MS4A4A	0.120851	0.01483
**TAM**	CCL2	0.144697	0.003478
	CD68	0.272391	2.44E-08
	IL10	0.110228	0.026354
**Neutrophils**	CD11b (ITGAM)	0.273472	2.14E-08
	CCR7	0.139655	9.40E-09
**Natural killer cell**	KIR2DL4	0.139655	0.004815
	KIR2DS4	0.106783	0.031466
	KIR3DL1	0.097806	0.04891
	KIR3DL2	0.211194	1.78E-05

## Conclusion

The study investigated the clinical value of the ARID1A in TCGA colon adenocarcinoma (COAD) tissues and cell lines and discovered that mir-185 downregulates ARID1A in COAD and that lower ARID1A expression was associated with high overall survival (OS), disease-free survival (DFS) (P<.05), and is an independent prognostic factor in colon cancer. Also, a correlational analysis was presented based on TCGA colon cancer data between mRNA ARID1A expression and clinicopathological features, tumor-infiltrated immune cells, immune inhibitors, activators, and chemokines.

## Data Availability Statement

The original contributions presented in the study are included in the article/**Supplementary Material**, further inquiries can be directed to the corresponding author/s.

## Author Contributions

SB conceived the ideas, design study, carried out the experiments, and wrote the manuscript. YQ, HW, ZZ, MZ, YG verified the analytical methods. Y-FZ supervised and review the manuscript. All authors contributed to the article and approved the submitted version.

## Conflict of Interest

The authors declare that the research was conducted in the absence of any commercial or financial relationships that could be construed as a potential conflict of interest.

## Publisher’s Note

All claims expressed in this article are solely those of the authors and do not necessarily represent those of their affiliated organizations, or those of the publisher, the editors and the reviewers. Any product that may be evaluated in this article, or claim that may be made by its manufacturer, is not guaranteed or endorsed by the publisher.

## Supplementary Material

Supplementary Figure 1 **(A)** volcano blot of ARID1A association results. **(B, C)** Top 50 positively and negatively associated ARID1A genes.

## References

[1] Wang H Liu J Li J Zang D Wang X Chen Y. Identification of Gene Modules and Hub Genes in Colon Adenocarcinoma Associated With Pathological Stage Based on WGCNA Analysis. Cancer Genet (2020) 242:1–7. 10.1016/j.cancergen.2020.01.052 32036224

[2] Kadoch C Hargreaves DC Hodges C Elias L Ho L Ranish J. Proteomic and Bioinformatic Analysis of Mammalian SWI/SNF Complexes Identifies Extensive Roles in Human Malignancy. Nat Genet (2013) 45:592–601. 10.1038/ng.2628 23644491 PMC3667980

[3] Zhang L Li Z Skrzypczynska KM Fang Q Zhang W O’Brien SA. Single-Cell Analyses Inform Mechanisms of Myeloid-Targeted Therapies in Colon Cancer. Cell (2020) 181:442–59.e29. 10.1016/j.cell.2020.03.048 32302573

[4] Shen J Peng Y Wei L Zhang W Yang L Lan L. Arid1a Deficiency Impairs the DNA Damage Checkpoint and Sensitizes Cells to PARP Inhibitors. Cancer Discovery (2015) 5:752–67. 10.1158/2159-8290.CD-14-0849 PMC4497871 26069190

[5] Ma K Araki K Ichwan SJA Suganuma T Tamamori-Adachi M Ikeda MA. E2FBP1/DRIL1, an AT-rich Interaction Domain-Family Transcription Factor, Is Regulated by P53. Mol Cancer Res (2003) 1:438–44. 12692263

[6] Wu SZ Roden DL Wang C Holliday H Harvey K Cazet AS. Stromal Cell Diversity Associated With Immune Evasion in Human Triple-Negative Breast Cancer. EMBO J (2020) 39:1–20. 10.15252/embj.2019104063 PMC7527929 32790115

[7] Kim YB Ahn JM Bae WJ Sung CO Lee D. Functional Loss of ARID1A is Tightly Associated With High PD-L1 Expression in Gastric Cancer. Int J Cancer (2019) 145:916–26. 10.1002/ijc.32140 30664822

[8] Mathur R Alver BH San Roman AK Wilson BG Wang X Agoston AT. ARID1A Loss Impairs Enhancer-Mediated Gene Regulation and Drives Colon Cancer in Mice. Nat Genet (2017) 49:296–302. 10.1038/ng.3744 27941798 PMC5285448

[9] Lee JH Ahn BK Baik SS Lee KH. Comprehensive Analysis of Somatic Mutations in Colorectal Cancer With Peritoneal Metastasis. In Vivo (Brooklyn) (2019) 33:447–52. 10.21873/invivo.11493 PMC6506280 30804124

[10] Wei XL Wang DS Xi SY Wu WJ Chen DL Zeng ZL. Clinicopathologic and Prognostic Relevance of ARID1A Protein Loss in Colorectal Cancer. World J Gastroenterol (2014) 20:18404–12. 10.3748/wjg.v20.i48.18404 PMC4277979 25561809

[11] Xie Z Li X He Y Wu S Wang S Sun J. Analysis of the Expression and Potential Molecular Mechanism of Interleukin-1 Receptor Antagonist (IL1RN) in Papillary Thyroid Cancer *Via* Bioinformatics Methods. BMC Cancer (2020) 20:1–13. 10.1186/s12885-020-07620-8 PMC7687764 33238942

[12] Lin Y Liang R Mao Y Ye J Mai R Gao X. Comprehensive Analysis of Biological Networks and the Eukaryotic Initiation Factor 4A-3 Gene as Pivotal in Hepatocellular Carcinoma. J Cell Biochem (2020) 121:4094–107. 10.1002/jcb.29596 31898336

[13] Zhang M Wang Y Wang Y Jiang L Li X Gao H. Integrative Analysis of DNA Methylation and Gene Expression to Determine Specific Diagnostic Biomarkers and Prognostic Biomarkers of Breast Cancer. Front Cell Dev Biol (2020) 8:529386. 10.3389/fcell.2020.529386 33365308 PMC7750432

[14] Lin Y Liang R Qiu Y Lv Y Zhang J Qin G. Expression and Gene Regulation Network of RBM8A in Hepatocellular Carcinoma Based on Data Mining. Aging (Albany NY) (2019) 11:423–47. 10.18632/aging.101749 PMC6366983 30670676

[15] Meng L Ding L Yu Y Li W. JAK3 and TYK2 Serve as Prognostic Biomarkers and Are Associated With Immune Infiltration in Stomach Adenocarcinoma. BioMed Res Int (2020) 2020:1. 10.1155/2020/7973568 PMC7559258 33083484

[16] Erfani M Hosseini SV Mokhtari M Zamani M Tahmasebi K Alizadeh Naini M. Altered ARID1A Expression in Colorectal Cancer. BMC Cancer (2020) 20:1–13. 10.1186/s12885-020-6706-x PMC7183124 32334542

[17] Yokota J. Tumor Progression and Metastasis. Carcinogenesis (2000) 21:497–503. 10.1093/carcin/21.3.497 10688870

[18] Lee LH Sadot E Ivelja S Vakiani E Hechtman JF Sevinsky CJ. ARID1A Expression in Early Stage Colorectal Adenocarcinoma: An Exploration of Its Prognostic Significance. Pathol (2017) 53:97–104. 10.1016/j.humpath.2016.02.004.ARID1A PMC4994515 26980037

[19] Yim SY Kang SH Shin JH Jeong YS Sohn BH Um SH. Low ARID1A Expression Is Associated With Poor Prognosis in Hepatocellular Carcinoma. Cells (2020) 9:1–14. 10.3390/cells9092002 PMC7564185 32878261

[20] Wang Dd Chen Yb Pan K Wang W Chen Sp Chen Jg. Decreased Expression of the ARID1A Gene Is Associated With Poor Prognosis in Primary Gastric Cancer. PloS One (2012) 7:e40364. 10.1371/journal.pone.0040364 PMC3396657 22808142

[21] Jiang X Wang J Deng X Xiong F Zhang S Gong Z. The Role of Microenvironment in Tumor Angiogenesis. J Exp Clin Cancer Res (2020) 39:1–19. 10.1186/s13046-020-01709-5 32993787 PMC7526376

[22] Morgensztern D. High TMB Predicts Immunotherapy Benefit. Cancer Discovery (2018) 8:668. 10.1158/2159-8290.CD-NB2018-048 29661758

[23] Roh W Chen P Reuben A Spencer CN Peter A Miller JP. Ntegrated Molecular Analysis of Tumor Biopsies on Sequential CTLA-4 and PD-1 Blockade Reveals Markers of Response and Resistance. Sci Transl Med (2018) 9:1–24. 10.1126/scitranslmed.aah3560.Integrated PMC5819607 28251903

[24] Fountzilas E Kotoula V Tikas I Manousou K Papadopoulou K Poulios C. Prognostic Significance of Tumor Genotypes and CD8+ Infiltrates in Stage I-III Colorectal Cancer. Oncotarget (2018) 9:35623–38. 10.18632/oncotarget.26256 PMC6235022 30479693

[25] Jung US Min KW Kim DH Kwon MJ Park H Jang HS. Suppression of arid1a Associated With Decreased Cd8 T Cells Improves Cell Survival of Ovarian Clear Cell Carcinoma. J Gynecol Oncol (2021) 32:1–13. 10.3802/jgo.2021.32.e3 PMC7767648 33185044

[26] Danaher P Warren S Dennis L D’Amico L White A Disis ML. Gene Expression Markers of Tumor Infiltrating Leukocytes. J Immunother Cancer (2017) 5:1–15. 10.1186/s40425-017-0215-8 28239471 PMC5319024

