# Supplementary figures and images for: Downregulated ARID1A by miR-185 Is Associated With Poor Prognosis and Adverse Outcomes in Colon Adenocarcinoma
Source: Front Oncol. 2021 Aug 2;11:679334. doi: 10.3389/fonc.2021.679334 (PMC8367751; doi:10.3389/fonc.2021.679334)

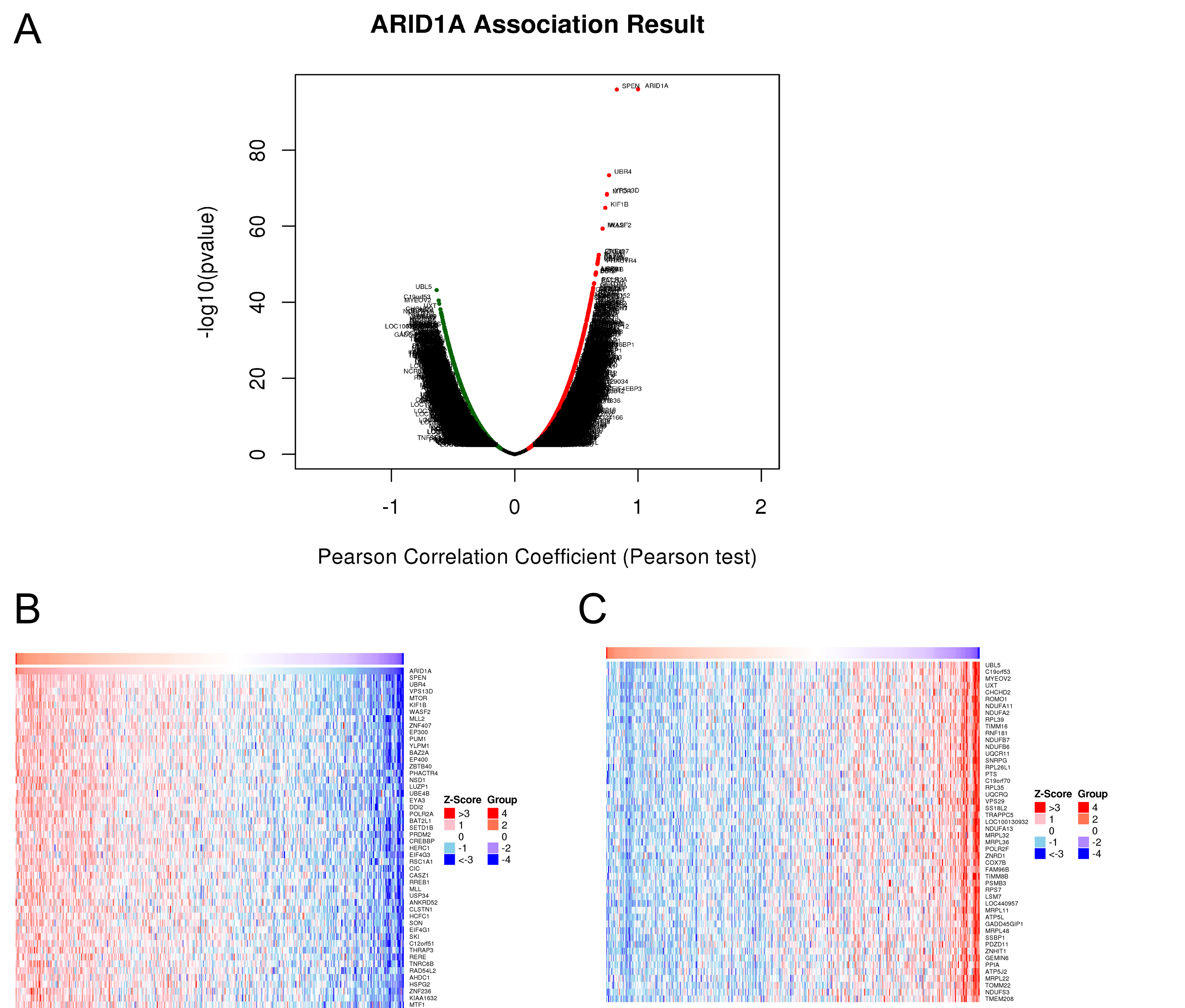

Supplement: Supplementary Figure 1 — (A) volcano blot of ARID1A association results. (B, C) Top 50 positively and negatively associated ARID1A genes. [file Image_1.tif]
