# Supplementary material for: Downregulated ARID1A by miR-185 Is Associated With Poor Prognosis and Adverse Outcomes in Colon Adenocarcinoma
Source: Front Oncol. 2021 Aug 2;11:679334. doi: 10.3389/fonc.2021.679334 (PMC8367751; doi:10.3389/fonc.2021.679334)
Supplement: Supplementary file 2 [file Table_1.docx]

ARID1A association with immunomodulators in COAD

| Immunomodulators | infiltrates | rho | P value |
| --- | --- | --- | --- |
| **chemokines** |  |  |  |
| COAD (n=458) | CCL11 | 0.138071 | 0.005323 |
| COAD (n=458) | CCL14 | 0.296427 | 1.12E-09 |
| COAD (n=458) | CCL18 | 0.135851 | 0.006114 |
| COAD (n=458) | CCL19 | 0.181779 | 0.000231 |
| COAD (n=458) | CCL2 | 0.144697 | 0.003478 |
| COAD (n=458) | CCL21 | 0.20088 | 4.57E-05 |
| COAD (n=458) | CCL22 | 0.241331 | 8.63E-07 |
| COAD (n=458) | CCL4 | 0.11888 | 0.016554 |
| COAD (n=458) | CCL5 | 0.210063 | 1.98E-05 |
| COAD (n=458) | CX3CL1 | 0.38645 | 6.54E-16 |
| COAD (n=458) | CXCL11 | 0.1103 | 0.026255 |
| COAD (n=458) | CXCL12 | 0.391503 | 2.54E-16 |
| COAD (n=458) | CXCL9 | 0.183354 | 0.000204 |
| **receptors** |  |  |  |
| COAD (n=458) | CCR1 | 0.247535 | 4.39E-07 |
| COAD (n=458) | CCR2 | 0.167401 | 0.000708 |
| COAD (n=458) | CCR4 | 0.268957 | 3.70E-08 |
| COAD (n=458) | CCR5 | 0.371272 | 1.03E-14 |
| COAD (n=458) | CCR6 | 0.109336 | 0.027604 |
| COAD (n=458) | CCR7 | 0.280077 | 9.40E-09 |
| COAD (n=458) | CCR8 | 0.390695 | 2.95E-16 |
| COAD (n=458) | CX3CR1 | 0.257558 | 1.42E-07 |
| COAD (n=458) | CXCR2P1 | 0.113459 | 0.022226 |
| COAD (n=458) | CXCR4 | 0.333164 | 5.57E-12 |
| COAD (n=458) | CXCR5 | 0.26834 | 3.99E-08 |
| COAD (n=458) | CXCR6 | 0.164268 | 0.000893 |
| COAD (n=458) | XCR1 | 0.247306 | 4.50E-07 |
| **stimulators** |  |  |  |
| COAD (n=458) | TNFRSF13B | 0.192956 | 9.12E-05 |
| COAD (n=458) | TNFRSF13C | 0.198442 | 5.67E-05 |
| COAD (n=458) | TNFRSF4 | 0.071725 | 0.149128 |
| COAD (n=458) | TNFRSF8 | 0.320418 | 3.81E-11 |
| COAD (n=458) | TNFRSF9 | 0.234075 | 1.86E-06 |
| COAD (n=458) | TNFSF13B | 0.194133 | 8.25E-05 |
| COAD (n=458) | TNFSF14 | 0.352851 | 2.39E-13 |
| COAD (n=458) | TNFSF15 | 0.367507 | 1.98E-14 |
| COAD (n=458) | TNFSF4 | 0.213938 | 1.37E-05 |
| COAD (n=458) | ULBP1 | 0.131653 | 0.007904 |
| COAD (n=458) | C10ORF54 | 0.262066 | 8.41E-08 |
| COAD (n=458) | CD27 | 0.149255 | 0.002569 |
| COAD (n=458) | CD28 | 0.298322 | 8.64E-10 |
| COAD (n=458) | CD40 | 0.110161 | 0.026446 |
| COAD (n=458) | CD40LG | 0.17439 | 0.000415 |
| COAD (n=458) | CD80 | 0.139628 | 0.004824 |
| COAD (n=458) | CD86 | 0.200761 | 4.62E-05 |
| COAD (n=458) | CXCL12 | 0.391503 | 2.54E-16 |
| COAD (n=458) | CXCR4 | 0.333164 | 5.57E-12 |
| COAD (n=458) | ENTPD1 | 0.321246 | 3.37E-11 |
| COAD (n=458) | ICOS | 0.170833 | 0.000546 |
| COAD (n=458) | ICOSLG | 0.393355 | 1.78E-16 |
| COAD (n=458) | IL2RA | 0.240749 | 9.18E-07 |
| COAD (n=458) | LTA | 0.191613 | 0.000102 |
| COAD (n=458) | MICB | 0.119048 | 0.0164 |
| COAD (n=458) | PVR | 0.381945 | 1.50E-15 |
| COAD (n=458) | TMEM173 | 0.275082 | 1.75E-08 |
| COAD (n=458) | TNFRSF13B | 0.192956 | 9.12E-05 |
| COAD (n=458) | TNFRSF13C | 0.198442 | 5.67E-05 |
| COAD (n=458) | TNFRSF14 | 0.150364 | 0.002384 |
| COAD (n=458) | ADORA2A | 0.20523 | 0.00003 |
| COAD (n=458) | BTLA | 0.25821 | 0.000000132 |
| COAD (n=458) | CD160 | 0.117572 | 0.01779155 |
| COAD (n=458) | CD274 | 0.239053 | 0.00000110 |
| COAD (n=458) | CD96 | 0.223287 | 0.00000555 |
| COAD (n=458) | CSF1R | 0.328745 | 0.0000000000110 |
| COAD (n=458) | CTLA4 | 0.294699 | 0.000000001 |
| COAD (n=458) | HAVCR2 | 0.166513 | 0.000756203 |
| COAD (n=458) | IDO1 | 0.13422 | 0.006761215 |
| COAD (n=458) | IL10 | 0.110228 | 0.026354119 |
| COAD (n=458) | KDR | 0.513025 | 0.00000000000000 |
| COAD (n=458) | LAG3 | 0.216349 | 0.0000109 |
| COAD (n=458) | LGALS9 | 0.161839 | 0.001065946 |
| COAD (n=458) | PDCD1 | 0.226264 | 0.0000041 |
| COAD (n=458) | PDCD1LG2 | 0.171743 | 0.000509494 |
| COAD (n=458) | PVRL2 | 0.345772 | 0.000000000000762 |
| COAD (n=458) | TGFB1 | 0.216456 | 0.0000108 |
| COAD (n=458) | TGFBR1 | 0.316653 | 0.0000000000660 |
| COAD (n=458) | TIGIT | 0.359764 | 0.0000000000000751 |
| COAD (n=458) | VTCN1 | 0.16466 | 0.000867413 |
